# Supplementary material for: Critical requirement of SOS1 for tumor development and microenvironment modulation in KRASG12D-driven lung adenocarcinoma
Source: Nat Commun. 2023 Sep 20;14:5856. doi: 10.1038/s41467-023-41583-1 (PMC10511506; doi:10.1038/s41467-023-41583-1)
Supplement: Supplementary file 1 — Supplementary Information [file 41467_2023_41583_MOESM1_ESM.pdf]

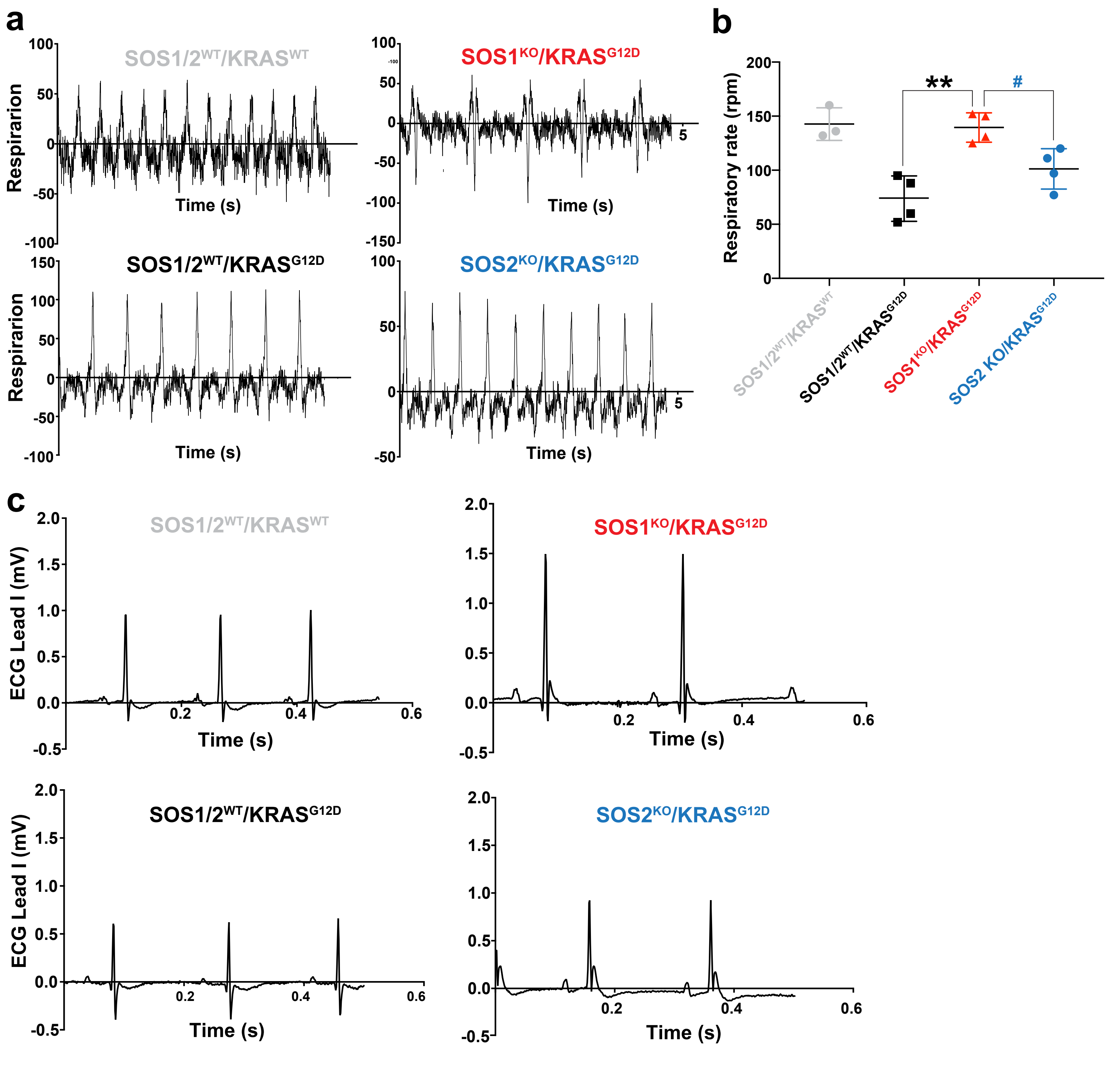
a
SOS1/2WT/KRASWT
SOS1KO/KRASG12D
SOS1/2WT/KRASG12D
SOS2KO/KRASG12D
Respirarion
Time (s)
b
Respiratory rate (rpm)
**
#
SOS1/2WT/KRASWT
SOS1/2WT/KRASG12D
SOS1KO/KRASG12D
SOS2 KO/KRASG12D
c
SOS1/2WT/KRASWT
SOS1KO/KRASG12D
SOS1/2WT/KRASG12D
SOS2KO/KRASG12D
ECG Lead I (mV)
Time (s)

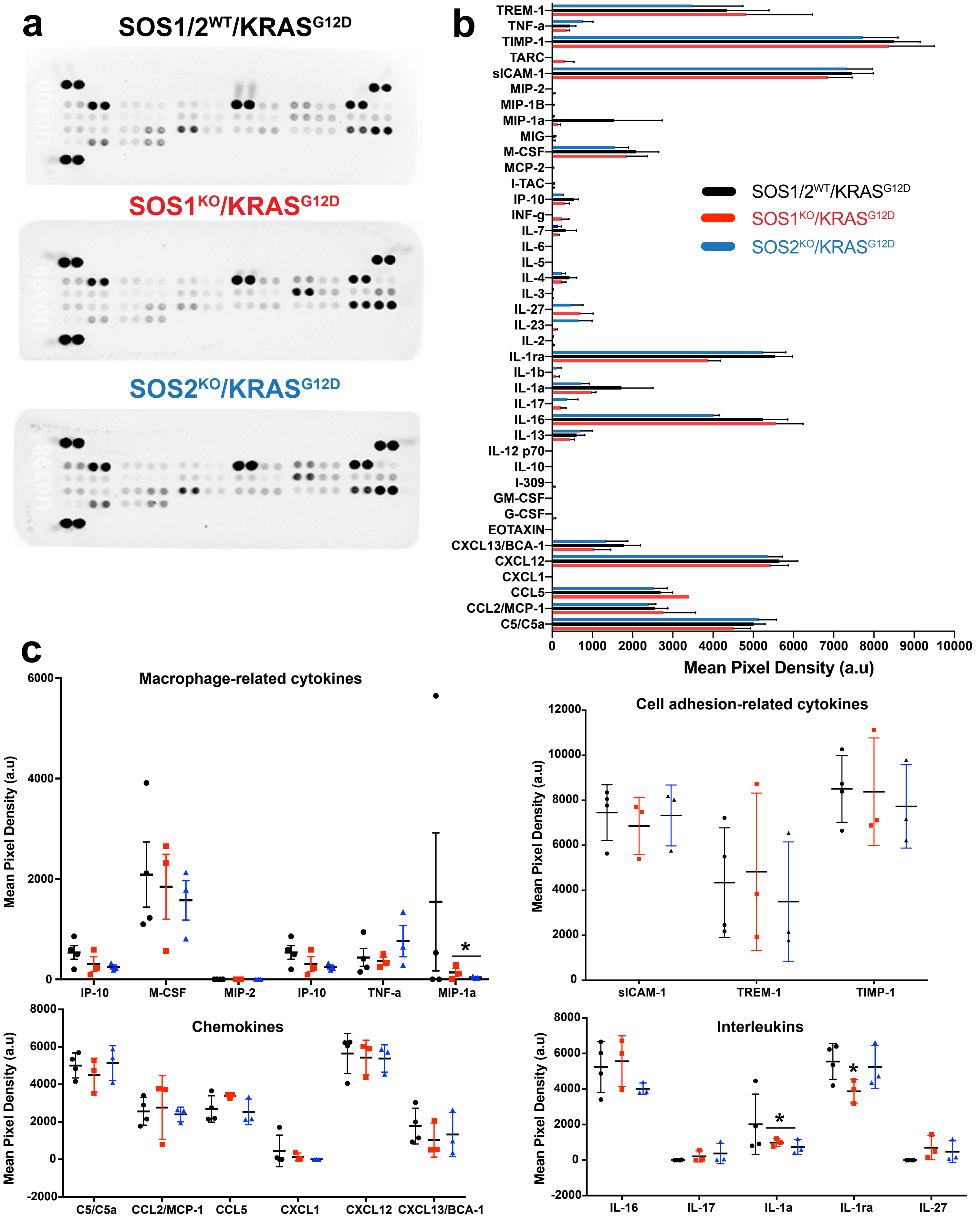

a
SOS1/2WT/KRASG12D
SOS1KO/KRASG12D
SOS2KO/KRASG12D
b
TREM-1
TNF-a
TIMP-1
TARC
sICAM-1
MIP-2
MIP-1B
MIP-1a
MIG
M-CSF
MCP-2
I-TAC
IP-10
INF-g
IL-7
IL-6
IL-5
IL-4
IL-3
IL-27
IL-23
IL-2
IL-1ra
IL-1b
IL-1a
IL-17
IL-16
IL-13
IL-12 p70
IL-10
I-309
GM-CSF
G-CSF
EOTAXIN
CXCL13/BCA-1
CXCL12
CXCL1
CCL5
CCL2/MCP-1
C5/C5a
Mean Pixel Density (a.u)
c
Macrophage-related cytokines
Mean Pixel Density (a.u)
IP-10
M-CSF
MIP-2
IP-10
TNF-a
MIP-1a
Cell adhesion-related cytokines
sICAM-1
TREM-1
TIMP-1
Chemokines
C5/C5a
CCL2/MCP-1
CCL5
CXCL1
CXCL12
CXCL13/BCA-1
Interleukins
IL-16
IL-17
IL-1a
IL-1ra
IL-27

Supplementary Figure S1. SOS1 depletion rescues LUAD-related pathophysiological parameters in KRAS^{G12D} mutated mice.

a-b, Representative respiratory profiles (**a**) and derived, color-coded graph plot (**b**) illustrating the respiratory rate in surviving, 12-month-old SOS1/2^{WT}/KRAS^{WT}, SOS1/2^{WT}/KRAS^{G12D}, SOS1^{KO}/KRAS^{G12D} and SOS2^{KO}/KRAS^{G12D} mice. Data expressed as mean ± SD. ***P*=0.0012 and #*P*=0.0439 vs SOS1^{KO}/KRAS^{G12D}. One-way ANOVA and Tukey's test. *n*=3 independent mice for SOS1/2^{WT}/KRAS^{WT} mice and *n*=4 independent mice for the rest of experimental groups. **c** Representative electrocardiogram (ECG) lead I recordings from 12-month-old mice of the indicated genotypes (SOS1/2^{WT}/KRAS^{WT}, SOS1/2^{WT}/KRAS^{G12D}, SOS1^{KO}/KRAS^{G12D} and SOS2^{KO}/KRAS^{G12D}). Source data are provided as a Source Data file.

Supplementary Figure S2. Impact of SOS1/2 ablation on pulmonary levels of cytokines and chemokines.

a Representative image of membrane arrays used for profiling 40 distinct cytokines and chemokines in lung tumor samples from KRAS^{G12D}-mutated, SOS1/2^{WT}, SOS1^{KO} and SOS2^{KO} mice. **b** Bar chart showing quantitation of the 40 molecular parameters of interest in each of the three color-coded experimental groups. **c,** Bar charts showing detailed quantitations grouped by specific families of cytokines and chemokines. Data shown as mean ± SD. *n*=3 independent samples per genotype. For MIP-1 (**P*=0.0488 vs SOS1^{KO} and **P*=0.0431 vs SOS2^{KO}). For IL-1ra (**P*=0.0234) vs SOS1/2^{WT}. One-way ANOVA and Tukey's test. C5a: Complement component 5a; CCL: C-C Motif Chemokine Ligand; CXCL: C-X-C motif chemokine; sICAM: Soluble intercellular adhesion molecule; IL: Interleukin; IP: Interferon-γ-Inducible Protein; MCP: Monocyte chemoattractant protein; M-CSF: Macrophage Colony stimulating factor; MIP: Macrophage Inflammatory Proteins; TIMP: TIMP Metallopeptidase Inhibitor; TNF: Tumor Necrosis Factor. TREM-1: triggering receptor expressed on myeloid cells. Source data are provided as a Source Data file.
