# Supplementary material for: Critical requirement of SOS1 for tumor development and microenvironment modulation in KRASG12D-driven lung adenocarcinoma
Source: Nat Commun. 2023 Sep 20;14:5856. doi: 10.1038/s41467-023-41583-1 (PMC10511506; doi:10.1038/s41467-023-41583-1)
Supplement: Supplementary file 3 — Reporting Summary [file 41467_2023_41583_MOESM3_ESM.pdf]

nature portfolio

Corresponding author(s): Fernando C. Baltanas and Eugenio Santos

Last updated by author(s): Aug 19, 2023

Reporting Summary

Nature Portfolio wishes to improve the reproducibility of the work that we publish. This form provides structure for consistency and transparency in reporting. For further information on Nature Portfolio policies, see our Editorial Policies and the Editorial Policy Checklist.

Statistics

For all statistical analyses, confirm that the following items are present in the figure legend, table legend, main text, or Methods section.

n/a	Confirmed	
☐	☒	The exact sample size (*n*) for each experimental group/condition, given as a discrete number and unit of measurement
☐	☒	A statement on whether measurements were taken from distinct samples or whether the same sample was measured repeatedly
☐	☒	The statistical test(s) used AND whether they are one- or two-sided<br>*Only common tests should be described solely by name; describe more complex techniques in the Methods section.*
☒	☐	A description of all covariates tested
☐	☒	A description of any assumptions or corrections, such as tests of normality and adjustment for multiple comparisons
☐	☒	A full description of the statistical parameters including central tendency (e.g. means) or other basic estimates (e.g. regression coefficient) AND variation (e.g. standard deviation) or associated estimates of uncertainty (e.g. confidence intervals)
☐	☒	For null hypothesis testing, the test statistic (e.g. *F*, *t*, *r*) with confidence intervals, effect sizes, degrees of freedom and *P* value noted<br>*Give P values as exact values whenever suitable.*
☒	☐	For Bayesian analysis, information on the choice of priors and Markov chain Monte Carlo settings
☒	☐	For hierarchical and complex designs, identification of the appropriate level for tests and full reporting of outcomes
☐	☒	Estimates of effect sizes (e.g. Cohen's *d*, Pearson's *r*), indicating how they were calculated

Our web collection on statistics for biologists contains articles on many of the points above.

Software and code

Policy information about availability of computer code

Data collection	DepMap (Cancer DependencyMap Portal, RRID:SCR_017655) identifies cancer vulnerabilities by identifying genetic dependencies in different types of human tumors. The specific gene dependency scores of hSOS1 and hSOS2 in human LUAD cell lines were calculated using the DepMap 22Q2 Public + Score dataset in their web portal (https://depmap.org/portal/, accessed on 5 February 2023).<br>The CANCERTOOL database and web-based computational tools (http://genomics.cicbiogune.es/CANCERTOOL/index.html, accessed 5 February 2023) was used for a comprehensive evaluation of the relevance of hSOS1 and hSOS2 gene expression data for LUAD development and survival
Data analysis	For quantification of western blot and immunochemical images, open-source ImageJ-win64 Fiji/ImageJ (version 1.53) was used.<br>GraphPad Prism (version 8.0.1, GraphPad Prism, RRID:SCR_002798) software was used to generate graphs and bar charts.<br>micro CT images were reconstructed and converted to 3D volumes using microCT Sedecal ACQ software and tumoral and non-tumoral segmentations were performed by using the 3D Slicer image computing platform (version 5.2.2).

For manuscripts utilizing custom algorithms or software that are central to the research but not yet described in published literature, software must be made available to editors and reviewers. We strongly encourage code deposition in a community repository (e.g. GitHub). See the Nature Portfolio guidelines for submitting code & software for further information.

Data

Policy information about availability of data

All manuscripts must include a data availability statement. This statement should provide the following information, where applicable:

- Accession codes, unique identifiers, or web links for publicly available datasets
- A description of any restrictions on data availability
- For clinical datasets or third party data, please ensure that the statement adheres to our policy

All data generated or analysed during this study are included in this published article (and its supplementary information files). CANCERTOOL database (http://genomics.cicbiogune.es/CANCERTOOL/) and DepMAP portal library (https://depmap.org/portal/) were used in this study.

Human research participants

Policy information about studies involving human research participants and Sex and Gender in Research.

Reporting on sex and gender	N/A
Population characteristics	N/A
Recruitment	N/A
Ethics oversight	N/A

Note that full information on the approval of the study protocol must also be provided in the manuscript.

Field-specific reporting

Please select the one below that is the best fit for your research. If you are not sure, read the appropriate sections before making your selection.

☒ Life sciences ☐ Behavioural & social sciences ☐ Ecological, evolutionary & environmental sciences

For a reference copy of the document with all sections, see nature.com/documents/nr-reporting-summary-flat.pdf

Life sciences study design

All studies must disclose on these points even when the disclosure is negative.

Sample size	No sample-size calculation was performed. Sample sizes were chosen based on the application of the 3Rs rules and our extended, previous experience characterizing and analyzing the SOS1/2 KO (Esteban et al., 2002; Baltanas et al., 2013, 2021; Liceras-Boillos et al., 2016, 2018; Garcia-Navas et al., 2021; Gomez et al., 2022) and the KRASG12D mouse colonies (Castellano et al., 2013, 2016). The minimum n value used for any experiment in this report was 3 (as indicated in each Figure legend). Notice also in this regard that, for example, the tumor volume measurements in Figure panel 1E were performed in 3 animals per genotype, and those data are complementary to the tumor burden data presented in Figure panel 1H, that was obtained from 3 different animals. Furthermore, 3 animals per genotype were also used in most experiments shown in Figures 2, 3, 4, 5 and 6, and we considered those cases that those n numbers were also adequate because our quantitative analyses always involved measurements of the total number of tumors present in all 5 lobes of the lungs of each animal analyzed, thus hugely increasing the actual, total n number of sample replicates per animal.
Data exclusions	KRASG12D mutant animals developing thymomas (<15%) were excluded from analysis.
Replication	n values mentioned in the figure legends indicate the number of animals used per experimental group. However, notice that our quantitative analyses always involved measurements of the total number of tumors present in all 5 lung lobes of each animal analyzed All attempts at replication were successful.
Randomization	Mice were allocated randomly (no gender selection) in the experimental groups.
Blinding	All experimental handlings by the investigators were blinded to group allocation during data collection and analysis.

Reporting for specific materials, systems and methods

We require information from authors about some types of materials, experimental systems and methods used in many studies. Here, indicate whether each material, system or method listed is relevant to your study. If you are not sure if a list item applies to your research, read the appropriate section before selecting a response.

Materials & experimental systems

n/a	Involved in the study
☐	☒ Antibodies
☐	☒ Eukaryotic cell lines
☒	☐ Palaeontology and archaeology
☐	☒ Animals and other organisms
☒	☐ Clinical data
☒	☐ Dual use research of concern

Methods

n/a	Involved in the study
☒	☐ ChIP-seq
☒	☐ Flow cytometry
☒	☐ MRI-based neuroimaging

Antibodies

Antibodies used

Rabbit anti-pERK1/2 (Cell Signaling, Cat# #9101).
Rabbit anti-Ki67 (Master Diagnostica, Cat# MAD-000310QD-3, clone SP6)
Mouse anti-SMA (Sigma-Aldrich, Cat# A5228, clone 1A4, purified from hybridoma cell culture).
Rabbit anti-cleaved-caspase 3 (CC3, Cell Signaling, Cat# #9579, clone D3E9)
Rabbit anti-CD68 (Abcam, Cat# ab125212)
Rabbit anti-CD3 (Abcam, Cat# ab5690).
Mouse anti-RAS (Millipore, Cat# 05-516, clone RAS10)
Mouse anti-SOS1 (BD Biosciences Cat#610096, RRID:AB_397502, Clone 25/SOS1)
Rabbit anti-SOS2 (Santa CruzBiotechnology, Cat# sc-15358,RRID:AB_2192446)
Mouse anti-beta-Tubulin (Sigma-Aldrich, Cat#T5293, RRID:AB_477580, clone 2-28-33, ascites fluid)
Rabbit anti-Vinculin (ProteinTech, Cat#26520-1-AP)
Goat anti-mouse DyLight 800 (ThermoFisher scientific, Cat#SA5-35521)
Goat anti-rabbit Fluor 680 (Invitrogen, Cat#A21076)

Validation

Rabbit anti-pERK1/2 (Cell Signaling, Cat# #9101).
The antibody does not cross-react with the corresponding phosphorylated residues of either JNK/SAPK or p38 MAP Kinase, and does not cross-react with non-phosphorylated Erk1/2.
1. Roux, P.P. and Blenis, J. (2004) Microbiol Mol Biol Rev 68, 320-44.
2. Baccarini, M. (2005) FEBS Lett 579, 3271-7.
3. Meloche, S. and Pouysségur, J. (2007) Oncogene 26, 3227-39.
4. Roberts, P.J. and Der, C.J. (2007) Oncogene 26, 3291-310.
5. Rubinfeld, H. and Seger, R. (2005) Mol Biotechnol 31, 151-74.
6. Murphy, L.O. and Blenis, J. (2006) Trends Biochem Sci 31, 268-75.
7. Dalby, K.N. et al. (1998) J Biol Chem 273, 1496-505.
8. Marais, R. et al. (1993) Cell 73, 381-93.
9. Kortenjann, M. et al. (1994) Mol Cell Biol 14, 4815-24.
10. Owens, D.M. and Keyse, S.M. (2007) Oncogene 26, 3203-13.

Rabbit anti-Ki67 (Master Diagnostica, Cat# MAD-000310QD-3, clone SP6).
IHC positive control: Tonsil. Visualization: Nuclear
1. Gerdes J, Lemke H, Baisch H, Wacker H-H, Schwab U, Stein H. Cell cycle analysis of a cell proliferationassociated human nuclear antigen defined by the monoclonal antibody Ki-67. J Immunol. 133:1710-5 (1984).
2. Gerdes J, Li L, Schlueter C, Duchrow M, Wohlenberg C, Gerlach C, Stahmer I, Kloth S, Brandt E, Flad HD. Immunobiochemical and molecular biologic characterization of the cell proliferation-associated nuclear antigen that isdefined by monoclonal antibody Ki-67. Am J Pathol. 138:867-73 (1991).
3. Cattoretti G, Becker MH, Key G, Duchrow M, Schlüter C, Galle J, Gerdes J. Monoclonal antibodies against recombinant parts of the Ki-67 antigen (MIB 1 and MIB 3) detect proliferating cells in microwave-processed formalinfixed paraffin sections. J Pathol. 168:357-63 (1992).
4. Key G, Becker MH, Baron B, Duchrow M, Schlüter C, Flad HD, Gerdes J. New Ki-67-equivalent murine monoclonal antibodies (MIB 1-3) generated against bacterially expressed parts of the Ki-67 cDNA containing three 62 base pair repetitive elements encoding for the Ki-67 epitope. Lab Invest 68:629-36 (1993).
5. Key G, Kubbutat MH, Gerdes J. Assessment of cell proliferation by means of an enzyme-linked immunosorbent assay based on the detection of the Ki-67 protein. J Immunol Methods 177:113-7 (1994).
6. Seshadri R, Leong AS-Y, McCaul K, Firgaira FA, Setlur V, Horsfall DJ. Relationship between p53 gene abnormalities and other tumour characteristics in breast-cancer prognosis [published erratum appears in Int J Cancer 1996;69:354]. Int J Cancer 69:135-41 (1996).
7. Borre M, Bentzen SM, Nerstrøm B, Overgaard J. Tumor cell proliferation and survival in patients with prostate cancer followed expectantly. J Urol 159:1609-14 (1998).
8. Huuhtanen RL, Blomqvist CP, Wiklund TA, Böhling TO, Virolainen MJ, Tukiainen EJ, Tribukait B, Andersson LC. Comparison of the Ki-67 score and S-phase fraction as prognostic variables in soft-tissue sarcoma. Br J Cancer 79:945-51 (1999).
9. Scholzen T, Gerdes J. The Ki-67 protein: from the known and the unknown [review]. J Cell Physiol 182:311-22 (2000).

Mouse anti-SMA (Sigma-Aldrich, Cat# A5228, clone 1A4, purified from hybridoma cell culture).
1.Skalli, O., et al., J. Cell Biol., 103, 2787-2796 (1986).
2.Abd-El-Basset, E., et al., Neurosci. Lett., 125, 117-120 (1991).
3.Durand-Arczynska, W., et al., Histochemistry, 100, 465-471 (1993).
4.van Royen, N., et al., FASEB, 16, 432-434 (2002).
5.Slaninova, I., et al., Antonie Van Leeuwenhoek., 75, 361-368 (1999).

6.Del Pup, L., et al., Int. J. Mol. Med., 10, 561-568 (2002)
Rabbit anti-CD68 (Abcam, Cat# ab125212)
Knockout validated. Positive control. IHC-P: Rat and mouse liver tissue, Mouse spleen, skin, and brain tissues; IHC-Fr: Rat liver tissue; WB: Wild-type RAW 264.7, Neuro-2a cell lysates, Rat and Mouse spleen tissue lysates. Raw264.7 cell lysate.
1. Kanai R et al. Interferon-? enhances the therapeutic effect of mesenchymal stem cells on experimental renal fibrosis. Sci Rep 11:850 (2021). PubMed: 33441701.
2. Saiyang X et al. Activation of Toll-like receptor 7 provides cardioprotection in septic cardiomyopathy-induced systolic dysfunction. Clin Transl Med 11:e266 (2021). PubMed: 33463061.
3. McClure MJ et al. RNU (Foxn1RNU-Nude) Rats Demonstrate an Improved Ability to Regenerate Muscle in a Volumetric Muscle Injury Compared to Sprague Dawley Rats. Bioengineering (Basel) 8:N/A (2021). PubMed: 33467489.
4. Nürnberger S et al. Repopulation of decellularised articular cartilage by laser-based matrix engraving. EBioMedicine 64:103196 (2021). PubMed: 33483297
5. Li B et al. Apelin/APJ relieve diabetic cardiomyopathy by reducing microvascular dysfunction. J Endocrinol 249:1-18 (2021).

Rabbit anti-cleaved-caspase 3 (CC3, Cell Signaling, Cat# #9579, clone D3E9)
1. Fernandes-Alnemri, T. et al. (1994) J Biol Chem 269, 30761-4.
2. Nicholson, D.W. et al. (1995) Nature 376, 37-43.

Rabbit anti-CD3 (Abcam, Cat# ab5690).
Positive and negative controls were made by the manufacturer and showed in the Datasheet.
1. Zhu X et al. HDAC1/2 Control Proliferation and Survival in Adult Epidermis and Pre?Basal Cell Carcinoma through p16 and p53. J Invest Dermatol 142:77-87.e10 (2022). PubMed: 34284046
2. Batra R et al. The sustained expression of Cas9 targeting toxic RNAs reverses disease phenotypes in mouse models of myotonic dystrophy type 1. Nat Biomed Eng 5:157-168 (2021). PubMed: 32929188
3. Longobardi C et al. Curcumin Modulates Nitrosative Stress, Inflammation, and DNA Damage and Protects against Ochratoxin A-Induced Hepatotoxicity and Nephrotoxicity in Rats. Antioxidants (Basel) 10:N/A (2021). PubMed: 34439487
4. Wilkinson H et al. PAR-1 signaling on macrophages is required for effective in vivo delayed-type hypersensitivity responses. iScience 24:101981 (2021). PubMed: 33458623
5. Morton JJ et al. Studying Immunotherapy Resistance in a Melanoma Autologous Humanized Mouse Xenograft. Mol Cancer Res 19:346-357 (2021).

Mouse anti-RAS (Millipore, Cat#05-516, clone RAS10)
This antibody has been reported to immunoprecipitate Ras.
1. Allen, M. P., et al. (2002). J. Biol. Chem. 277: 38133-40.
2. Yusoff, P., et al. (2002). J. Biol. Chem. 277: 3195-201.
3. Okano, J., et al. (2001). J. Biol. Chem. 276: 19555-64.
4. Hamer, P. J., et al. (1990). Hybridoma. 9: 573-87

Mouse anti-SOS1 (BD Biosciences Cat#610096, RRID:AB_397502, Clone 25/SOS1)
Knockout or CRISPR-mediated SOS1 deletion validated by western-blot by the authors.
1. Buday L, Downward J. Epidermal growth factor regulates p21ras through the formation of a complex of receptor, Grb2 adapter protein, and Sos nucleotide exchange factor. Cell. 1993; 73(3):611-620. (Biology).
2. Egan SE, Giddings BW, Brooks MW, Buday L, Sizeland AM, Weinberg RA. Association of Sos Ras exchange protein with Grb2 is implicated in tyrosine kinase signal transduction and transformation. Nature. 1993; 363(6424):45-51. (Biology).
3. Furuta S, Miura K, Copeland T, Shang WH, Oshima A, Kamata T. Light Chain 3 associates with a Sos1 guanine nucleotide exchange factor: its significance in the Sos1-mediated Rac1 signaling leading to membrane ruffling. Oncogene. 2002; 21(46):7060-7066.
4. Kardinal C, Konkol B, Lin H, et al. Chronic myelogenous leukemia blast cell proliferation is inhibited by peptides that disrupt Grb2-SoS complexes. Blood. 2001; 98(6):1773-1781.
5. Salojin KV, Zhang J, Meagher C, Delovitch TL. ZAP-70 is essential for the T cell antigen receptor-induced plasma membrane targeting of SOS and Vav in T cells. J Biol Chem. 2000; 275(8):5966-5975..

Rabbit anti-SOS2 (Santa CruzBiotechnology Cat# sc-15358,RRID:AB_2192446)
Knockout or CRISPR-mediated SOS2 deletion validated by western-blot by the authors
1. Baltanas et al. Functional Specificity of the Members of the Sos Family of Ras-GEF Activators: Novel Role of Sos2 in Control of Epidermal Stem Cell Homeostasis. Cancers. 2021.
2. Liceras-Boillos et al. Differential Role of the RasGEFs Sos1 and Sos2 in Mouse Skin Homeostasis and Carcinogenesis. Mol Cell Biol. 2018.
3. Kortum et al. Deconstructing Ras signaling in the thymus. Mol Cell Biol. 2012.

Mouse anti-beta-Tubulin (Sigma-Aldrich Cat#T5293, RRID:AB_477580, clone 2-28-33, ascites fluid).
Antibody enhanced validation.
1. Siddiqui, S. S., et al., J. Neurosci., 9, 2963 (1989).
2. Crowther, R. J., and Whittaker, J. R., J. Neurobiol.,23, 280 (1992).
3. Joshi, H. C., and Cleveland, D. W., Cell. Motil.Cytoskeleton, 16, 159 (1990).
4. Banerjee, A., et al., J. Biol. Chem., 265, 1794(1990).
5. Piperno, G., et al., J. Cell Biol., 104, 289 (1987)

Rabbit anti-Vinculin (ProteinTech, Cat#26520-1-AP)
1. Chen et al., NPRL2 enhances autophagy and the resistance to Everolimus in castration-resistant prostate cancer.
2. Li et a., Targeting glutaminase 1 attenuates stemness properties in hepatocellular carcinoma by increasing reactive oxygen species and suppressing Wnt/beta-catenin pathway.
3. Li et al., Zyxin-involved actin regulation is essential in the maintenance of vinculin focal adhesion and chondrocyte differentiation status.

Eukaryotic cell lines

Policy information about cell lines and Sex and Gender in Research

Cell line source(s)	HEK-293-T cells were originally from the ATCC. The KPB6 cells were generated in Sergio Quezada´s Laboratory (University College London), used by Julian Downward's lab (Coelho et al., 2017) and kindly provided by Dr. Esther Castellano. The LKR10 and LKR13 are mouse lung cancer cell lines derived by serial passage of minced lung adenocarcinoma tissues from two tumors isolated from separate lobes of the KrasLA1 mouse model (Johnson et al., 2001) and gently provided by Dr. Esther Castellano.
Authentication	HEK-293-T cells were authenticated by ATCC (STR profiling). KPB6, LKR10 and LKR13 cell lines were not authenticated.
Mycoplasma contamination	We confirm that all cell lines used (KPB6, LKR10, LKR13 and HEK293T) tested negative for mycoplasma contamination
Commonly misidentified lines (See ICLAC register)	none

Animals and other research organisms

Policy information about studies involving animals; ARRIVE guidelines recommended for reporting animal research, and Sex and Gender in Research

Laboratory animals	The KRASLA2 mouse strain spontaneously developing KRASG12D-driven LUAD (Johnson et al., 2001) was crossmated to our tamoxifen (TMX)-inducible SOS1/2KO mouse system (Baltanas et al., 2013) to generate KRASG12D-expressing mice of the relevant SOS genotypes (SOS1/2WT, SOS1KO, SOS2KO). Mice (Mus musculus; no gender selected) kept in an homogenous C57BL/6J background (RRID:IMSR_JAX:000664), from 1 month to 12 months of age, were used. The animals were housed in cages with adequate space, bedding material for comfort and maintained under specific pathogen free conditions, while maintaining 12-hour dark/light. The ambient temperature was kept within 20-24[illegible]C, and humidity levels ranged from 45-65%.
Wild animals	The study did not involve wild animals
Reporting on sex	Sexual differences were not considered as a biological variable
Field-collected samples	The study did not involve samples collected from the field
Ethics oversight	Mice were kept, managed and sacrificed in the NUCLEUS animal facility of the University of Salamanca according to European (2007/526/CE) and Spanish (RD1201/2005 and RD53/2013) legislations. All experiments were approved by the Bioethics Committee of the Cancer Research Center (#596)

Note that full information on the approval of the study protocol must also be provided in the manuscript.
